# Permethrin exposure primes neuroinflammatory stress response to drive depression-like behavior through microglial activation in a mouse model of Gulf War Illness

**DOI:** 10.1186/s12974-024-03215-3

**Published:** 2024-09-13

**Authors:** Sean X. Naughton, Eun-Jeong Yang, Umar Iqbal, Kyle Trageser, Daniel Charytonowicz, Sibilla Masieri, Molly Estill, Henry Wu, Urdhva Raval, Weiting Lyu, Qing-li Wu, Li Shen, James Simon, Robert Sebra, Giulio Maria Pasinetti

**Affiliations:** 1 Department of Neurology, Icahn School of Medicine at Mount Sinai, New York, NY USA; 2 Department of Genetics and Genomic Sciences, Icahn School of Medicine at Mount Sinai, New York, NY USA; 3 Department of Neuroscience, Icahn School of Medicine at Mount Sinai, New York, NY USA; 4 Department of Plant Biology, Rutgers University, New Brunswick, NJ USA; 5 Geriatric Research, Education and Clinical Center, James J. Peters Veterans Affairs Medical Center, Bronx, NY USA

## Abstract

Gulf War Illness (GWI) is a chronic multisymptom disorder that affects approximately 25–32% of Gulf War veterans and is characterized by a number of symptoms such as cognitive impairment, psychiatric disturbances, chronic fatigue and gastrointestinal distress, among others. While the exact etiology of GWI is unknown, it is believed to have been caused by toxic exposures encountered during deployment in combination with other factors such as stress. In the present study we sought to evaluate the hypothesis that exposure to the toxin permethrin could prime neuroinflammatory stress response and elicit psychiatric symptoms associated with GWI. Specifically, we developed a mouse model of GWI, to evaluate the effects of chronic permethrin exposure followed by unpredictable stress. We found that subjecting mice to 14 days of chronic permethrin exposure followed by 7 days of unpredictable stress resulted in the development of depression-like behavior. This behavioral change coincided with distinct alterations in the microglia phenotype, indicating microglial activation in the hippocampus. We revealed that blocking microglial activation through Gi inhibitory DREADD receptors in microglia effectively prevented the behavioral change associated with permethrin and stress exposure. To elucidate the transcriptional networks impacted within distinct microglia populations linked to depression-like behavior in mice exposed to both permethrin and stress, we conducted a single-cell RNA sequencing analysis using 21,566 single nuclei collected from the hippocampus of mice. For bioinformatics, UniCell Deconvolve was a pre-trained, interpretable, deep learning model used to deconvolve cell type fractions and predict cell identity across spatial datasets. Our bioinformatics analysis identified significant alterations in permethrin exposure followed by stress-associated microglia population, notably pathways related to neuronal development, neuronal communication, and neuronal morphogenesis, all of which are associated with neural synaptic plasticity. Additionally, we observed permethrin exposure followed by stress-mediated changes in signal transduction, including modulation of chemical synaptic transmission, regulation of neurotransmitter receptors, and regulation of postsynaptic neurotransmitter receptor activity, a known contributor to the pathophysiology of depression in a subset of the hippocampal pyramidal neurons in CA3 subregions. Our findings tentatively suggest that permethrin may prime microglia towards a state of inflammatory activation that can be triggered by psychological stressors, resulting in depression-like behavior and alterations of neural plasticity. These findings underscore the significance of synergistic interactions between multi-causal factors associated with GWI.

**Supplementary Information:**

The online version contains supplementary material available at 10.1186/s12974-024-03215-3.

## Introduction

A significant proportion of veterans who have experienced deployment face the debilitating effects of depression upon their return to civilian life [1]. Several studies indicate that the prevalence of depression is notably higher among deployed veterans compared to the general population, underscoring the urgent need for targeted interventions and support services to address the mental health needs of this vulnerable group [2]. Following their return from deployment, veterans initially appeared healthy [3]. Still, over time, various health problems emerged, with research identifying traumatic brain injury, social isolation, and psychological stress as key factors contributing to the development and persistence of depressive symptoms [3, 4]. Understanding the complex interplay between deployment-related stressors and depressive symptomatology is crucial for developing effective prevention and treatment strategies tailored to the unique needs of this demographic.

Gulf War Illness (GWI) is a term used to describe a range of chronic and often debilitating symptoms reported by veterans who served in the 1990–1991 Gulf War (GW). At least 25% of Gulf War veterans have reported symptoms such as significant mood disturbances and neurological issues, fatigue, musculoskeletal pain, cognitive problems, respiratory difficulties, gastrointestinal issues, and rashes [5]. Exposure to GW-related chemicals, such as pyridostigmine bromide (a drug used to protect against nerve agents like sarin gas), N, N-diethyl-m-toluamide, and pyrethroid insecticides, has been linked to the development of GWI [6]. Pyrethroids, including permethrin, were applied by soldiers to their skin and uniforms every 4–5 days using a 0.5% spray, with reported usage significantly exceeding the recommended guidelines [7]. Permethrin exposure is currently recognized as a potential factor contributing to the onset of GWI [8, 9]. The primary mechanism of action for permethrin involves binding to voltage-gated sodium channels (VGSCs), leading to prolonged opening and alterations in neuronal firing [10–12]. Previous studies have shown that pyrethroids, which can cross the blood-brain barrier (BBB), can stimulate microglial activation by interacting with VGSCs in microglia [13]. This interaction can lead to an excessive accumulation of intracellular sodium ions and the release of major pro-inflammatory cytokines, such as tumor necrosis factor-alpha in microglia [13].

The delay between their deployment and the onset of persistent fatigue, problems, affective psychological disorders such as depression, and cognitive dysfunctions, among others, has made it difficult for the medical community to target a common underlying mechanism primarily related to the mental health of the disorder accurately [3]. Clinical research has recently reported that immune dysfunction and chronic inflammation play significant roles in the symptoms experienced by affected veterans [14]. Both the central nervous system and peripheral immune system are implicated in the pathogenesis of GWI, as observed in rodent models [15]. Microglia play a crucial role in neuroinflammation by initiating a range of inflammatory responses upon activation, including the release of pro-inflammatory cytokines and alterations to the neuronal environment [16]. In particular, microglia have been observed to adapt their morphology and function to support neurons through various mechanisms: releasing soluble factors that modulate neurotransmission, phagocytosing damaged dendritic elements, and facilitating synaptic plasticity [17]. This dynamic interaction between microglia and neurons has been increasingly recognized as a critical factor in the onset and progression of depression [18]. Hence, a comprehensive understanding of the molecular mechanisms behind these immune responses in the brain under GW-related conditions is vital for advancing the development of biomarkers and therapies that address symptoms and neuropathology of GWI, especially about depression.

The impact of prolonged exposure to permethrin, particularly when combined with GW-related chemicals, has been shown to affect neurological outcomes and other CNS-related symptoms in preclinical studies [19]. In order to better understand the nature of these neurological changes associated with exposure to GWI, it is essential to develop models that not only replicate the diverse chemicals and conditions of GW deployment but also account for potential interactions among physiological and psychological stressors. Thus, a comprehensive approach is crucial to identify causal factors and develop effective therapeutic strategies. In the present study, we sought to simulate GWI-like conditions in mice by administering permethrin followed by unpredictable stress to evaluate the combined effects on neuroinflammation caused by microglial activation, neural activity, and neurophysiological impairments and to uncover the underlying mechanistic factors in GWI.

## Materials and methods

### Animals

All of the experimental procedures were approved by the Animal Care Committee of Icahn School of Medicine at Mount Sinai (Approval number: IACUC-2019-0043). Mice, including C57BL/6 (WT), Cx3Cr1^CreEr^ (strain: 021160), and hM4Di (Designer Receptors Exclusively Activated by Designer Drugs (DREADD), R26-hM4Di/mCitrine, (strain: 026219)), were procured from the Jackson Laboratory. Crossbreeding was conducted between Cx3Cr1^CreEr^ mice and transgenic mice expressing cre recombinase-inducible hM4Di-DREADD, aiming to establish consistent expression of Gi inhibitory receptors on microglia [20]. Double transgenic mice were identified by PCR as recommended by Jackson Laboratory, while nontransgenic littermates were employed as age-matched controls. For genotyping, the primers were used (Cx3Cr1^CreEr^;mutant reverse; 5′-CGGTTATTCAACTTGCACCA-3′, WT reverse; 5′-GGATGTTGACTTCCGAGTTG − 3′, and common forward; 5′-AAGACTCACGTGGACCTGCT-3′, R26-hM4Di/mCitrine; mutant reverse; 5′-TCATAGCGATTGTGGGATGA-3′, mutant forward; 5′-CGAAGTTATTAGGTCCCTCGAC-3′, WT reverse; 5′-CCGAAAATCTGTGGGAAGTC-3′, and WT forward; 5′-AAGGGAGCTGCAGTGGAG TA-3. WT mice were used in all experiments except for Fig. 2D, which specifically displays data from Cx3Cr1^CreEr^/hM4Di-DREADD mice. Each cage (*N* = 4–5 mice) was housed under a 12-hour light/dark cycle, with lights on from 07:00 to 19:00 h, maintaining a steady temperature of 23 °C in a room with water and a standard rodent chow diet (LabDiet, MO, USA,5053). They had unrestricted access to food and water. The Institutional Animal Care and Use Committee (approval number: IACUC-2019-0043) at the Icahn School of Medicine at Mount Sinai approved all protocols that complied with NIH guidelines. Female mice were excluded from this study because stress-induced behavioral deficits vary by sex and require different behavioral paradigms to produce comparable stress-induced behavioral and immunological outcomes [21]. As a result, the conclusions of this study are limited to male mice.

### Permethrin pharmacokinetic studies

C57BL/6J mice (*N* = 5 per each group) were administered an acute dose of 200 mg/kg permethrin and sacrificed at 0, 2, 4, 18, 24, 28, 30, or 48 h post-treatment. Additionally, an untreated control group was included to validate analytical detection. Blood was collected via cardiac puncture, and the animals were immediately perfused with ice-cold PBS. Brains were then collected and homogenized in 0.2% formic. All blood and homogenized tissue samples were promptly snap-frozen and stored at -80 °C until further processing.

#### Analytical method

Three internal standards (ISs), 13C6-trans-permethrin (50 µg/mL), 13C6-cis-permethrin (50 µg/mL), and 13C6-3-phenoxybenzoic acid (100 µg/mL), were dissolved in nonane and mixed, then diluted in acetone to make the final concentration of 13C6-trans-permethrin, 13C6-cis-permethrin, and 13C6-3-phenoxybenzoic acid was 33 ng/mL, 33 ng/mL, and 67 ng/mL, respectively. The combined organic phases were evaporated to dryness under nitrogen before reconstitution for LC-MS/MS analysis. The residue was reconstituted in 400 µL of acetonitrile and centrifuged at 16,500 xg for 10 min. For each sample extract, 1 µL was injected into an UPLC-QqQ/MS system for analysis under dynamic multiple reaction monitoring mode in duplicate.

#### LC-MS method

The instrument used for chemical analysis was an Agilent 1290 Infinity II UHPLC (Agilent Technology, CA, USA) hyphenated with 6470 triple quadrupole mass spectrometry with electrospray ionization source (ESI) (Santa Clara, CA, USA). Agilent MassHunter Optimizer (version B.07.00) was used for standard compound-related parameters optimization, and MassHunter Workstation software Data Acquisition (version B.08.00) and Quantitative Analysis (version B.07.01) were used for data processing. The columns used for compound separation were AcquityTM Premier HSS T3 C18 VanGuardTM FIT (2.1 × 100 mm, 1.8 μm). For the chromatographic part, mobile phase A was water, and mobile phase B was methanol; both mobile phases were modified with 10 mM ammonium acetate. The compounds were separated using a gradient program. The mass spectrometer operated in the positive ionization mode for permethrin and the negative ionization mode for 3-PBA. WinNonlin professional software version 8.0 (Pharsight, CA, USA) was used to calculate the pharmacokinetic parameters. The peak plasma or brain concentration (C_max_) and the peak time (T_max_) were expressed as the mean ± SEM.

### Animal treatments

As previously established for permethrin exposure [19, 22], mice aged 7–8 weeks of the C57/BL6J strain (*N* = 9–10 per group) were randomly allocated to treatment cohorts. They were administered either permethrin (200 mg/kg in 5% DMSO in corn oil, Sigma-Aldrich, MO, USA) or vehicle (5% DMSO in corn oil) via intraperitoneal injection for 14 days. Following this treatment period, mice were exposed to unpredictable or no stress for 7 days. Behavioral testing took place on the subsequent day (day 22), followed by euthanasia of the animals on the same day. To inhibit microglia activation, Cx3Cr1^CreEr^/hM4Di-DREADD mice were treated with JHU37160 (Tocris Bioscience, Bristol, UK, 7198/50) for 14 consecutive days.

### Unpredictable stress exposure

Consistent with our ongoing study and published experiment [23], mice were exposed to random, unpredictable stress for 7 days, which is insufficient to induce depression-like behavior. The unpredictable stress paradigm involved subjecting animals to two stressors daily, administered separately in the morning (10:00–11:00) and evening sessions (17:00–18:00) (Table 1). Briefly, stressors included wet bedding for 6 h, placing cages on an orbital shaker for 20 min, exposure to hot air (via a hair dryer) for 10 min, tilting cages at a 45° angle for overnight, forced restraint for 60 min, cold water exposure for 5 min, no bedding for 6 h, overnight water restriction, and exposure to light during the dark cycle.

**Table 1:**
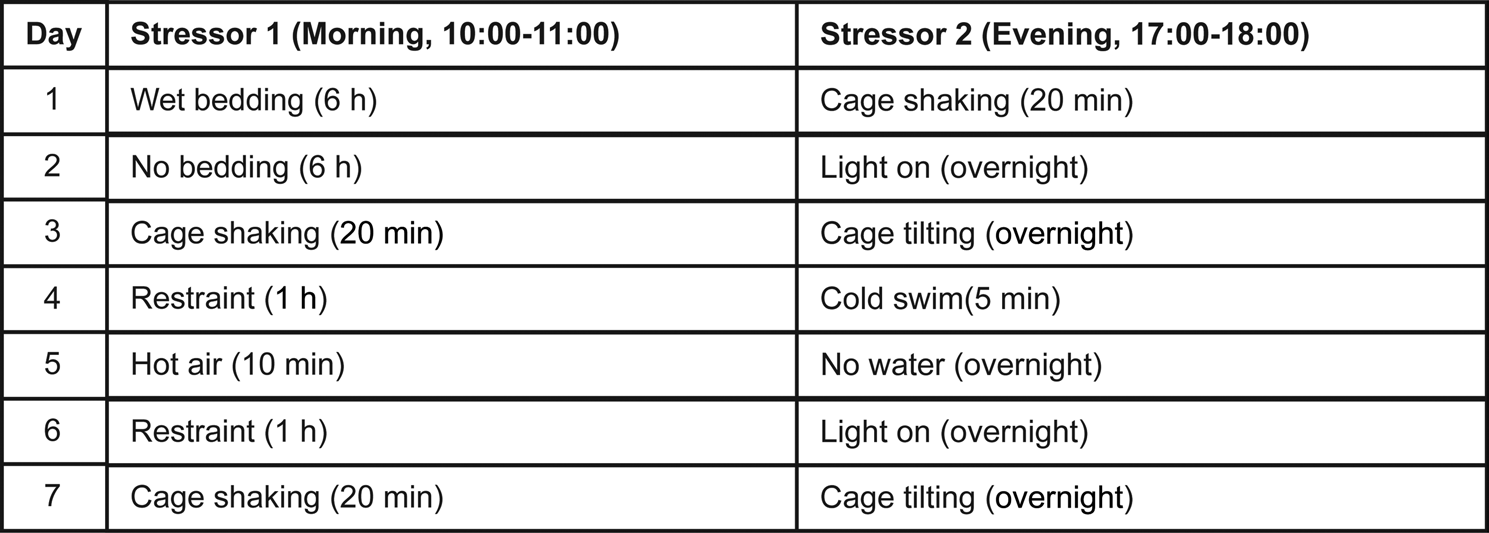
Stress schedule

### Behavioral tests

Before initiating behavioral testing, all animals were introduced into the behavioral testing room and given one hour to acclimate in their respective home cages. We performed the Open Field Test (OFT) first, followed by the Forced Swim Test (FST) on the same day. A rest period of 2 h was provided between the two behavioral assessments. The behavioral session was recorded via a near-infrared camera and analyzed using ANY-maze™ tracking software (Stoelting Co., IL, USA). Each test employed established protocols, detailed below:

#### OFT

As previously established [24], mice(*N* = 9–10 per group) were positioned within a plastic square enclosure measuring 40 cm × 40 cm × 38 cm for 15 min to measure anxiety-like behavior. The behavioral session was captured on the video camera and analyzed using ANY-maze™ software. Quantification of anxiety-like behavior involved measuring the total time spent along the edges and corners of the box, as well as the time spent in the center of the open field.

#### FST

As previously established [25], mice (*N* = 9–10 per group) were introduced into a large 4 L beaker filled with clean water at approximately 21 °C for 6 min to assess depressive-like behavior. The total duration of immobility, defined as the absence of movement in all four limbs, was measured using ANY-maze software.

### Tissue preparation

After completing the exposure paradigm and behavioral testing, mice were randomly assigned for use in immunohistochemistry or molecular studies. For Immunohistochemistry studies, mice were euthanized by administering 100 mg/kg ketamine and 10 mg/kg xylazine, followed by intracardiac perfusion with 1× phosphate-buffered saline (PBS), followed by perfusion with 4% paraformaldehyde (PFA). The brain tissue was fixed in 4% PFA for 24 h for subsequent immunohistochemistry. For mice used in molecular studies mice were euthanized by administering 100 mg/kg ketamine and 10 mg/kg xylazine, followed by thoracotomy. Brain tissues, were dissected into the hippocampus for single-nucleus sequencing or Olink assays. All samples were stored at − 80 °C–4 °C until further analysis.

### Immunohistochemistry

Fixed brains (*N* = 4–6 per group) were immersed in 4% PFA and subsequently dehydrated in PBS. Brain Section (50 μm) underwent washing with PBS, permeabilization with PBS + 0.2% Triton X-100 (PBST), and blocking with 2% normal goat serum in PBST. Ionized calcium-binding adaptor molecule 1(IBA-1,1:1000, Ab178846, Abcam, MA, USA) was applied and incubated overnight at 4 °C in PBST with 2% normal goat serum. Following rinsing, brain sections were treated with Alexa Fluor 568-labeled (1:500 in 2% normal goat serum in PBST) for 1 h at room temperature. Brain sections were then washed, mounted, and coverslipped on microscope slides. Imaging was conducted using a Zeiss LSM 880 Confocal Microscope (Zeiss, DE, Germany). Microscopy and image analysis were performed at the Microscopy CoRE at the Icahn School of Medicine at Mount Sinai.

### Sholl analysis

To assess the complexity of each cell, a Sholl analysis was conducted [26]. The determination of soma volume, branch length, number of terminal points, and number of intersection segments in brain sections from the hippocampus and prefrontal cortex (PFC) was conducted utilizing IMARIS 9.1.2 (Bit Plane Inc, MA, USA). The analysis encompassed the following steps: In 3D analysis, z-stack confocal images underwent processing using AutoQuant X3.1 (Media Cybernetics, MD, USA) to eliminate blurriness, followed by analysis with IMARIS. The surface tool was utilized to reconstruct the cell bodies (somas) of the microglia, whereas the filaments tool was employed to reconstruct the branches. One microglial cell was chosen per total process area, and its cell body and soma were traced along with the surface and filament for each process. The quantification involved determining the number of primary processes and branch tips. Each image selected one microglial cell per 0.045 µm^2^, with concentric circles drawn from the soma at 5 μm spacing to measure the intersections of each cell with each circle. Soma volume, branch length, number of terminal points, and number of intersection segments were quantified, and averages were determined among samples within the same group. The analysis encompassed 4 to 6 mice (2–4 tissue sections per mouse and 4–10 microglia per section) per group, conducted by a single investigator, while an independent evaluator performed the quantification analysis in a blinded manner, utilizing code to conceal knowledge of the experimental groups.

### Single nuclei sequencing (scRNA-Seq)

Single nuclei suspensions were processed using the 10X Genomics Chromium NEXT Gem Single Cell 3’ v3.1 kit, targeting 6000 cells per sample (*N* = 1 per group). The resulting libraries were sequenced on a NovaSeq 6000, targeting 50,000 reads per single nucleus. Sequenced reads were processed and aligned to < Genome > using the 10X Genomics *Cellrange*r toolkit. The resulting raw counts matrices were further preprocessed using the *scanpy 1.8.2* package. In brief, raw unique molecular identifier (UMI) count matrices for four experimental samples were passed through *cellbender 0.2.0*, for the purposes of filtering out empty droplets and reducing background noise stemming from ambient RNA. The relevant parameter settings for each sample for *cellebender* as follow; Vehicle-no stress; expected cell:10,000;Total Droplets Included:20,000; False Positive Rate:0.1; Epochs:100, Vehicle-stress; expected cell:12,434;Total Droplets Included:15,000; False Positive Rate:0.01; Epochs:100, Permethrin-no stress; expected cell:4212;Total Droplets Included:10,000; False Positive Rate:0.01; Epochs:100, Permethrin-stress; expected cell:3368;Total Droplets Included:10,000; False Positive Rate:0.01; Epochs:100. Following successful droplet filtering and ambient RNA removal, *scrublet 0.2.3* was used with default configuration parameters to detect and remove cellular doublets. Quality control metrics were calculated for each cell and gene across all samples, including the calculation of percentage of count deriving from mitochondrial (mt), hemoglobin (hb), and ribosomal (ribo) genes (Supplementary Fig. 1). On a per-sample basis, outlier cells were identified on the basis of any one of the following metrics falling outside 5-times the median absolute deviation: *log1p_total_counts*,* log1p_n_genes_by_counts*,* pct_counts_in_top_20_genes*,* pct_counts_mt*,* pct_counts_hb*, and *pct_counts_ribo*. Lastly, genes expressed in less than 20 cells were filtered out. In total, 21,566 cells and 21,545 genes remained after filtering. Total counts per cell were normalized to 1e^4^, with data scaled to log-plus-one. The scanpy function *sc.pp.highly_variable_genes* was used to calculate highly variable genes, with a minimum mean of 0.0125, a maximum mean of 3, and a minimum dispersion of 0.5. The effects of *pct_counts_mt* and *total_counts* were regressed out using the scanpy function *sc.pp.regress_out*. Following this, each genes expression was re-scaled to zero mean and one standard deviation. Dimensionality reduction was initiated utilizing Principle Components Analysis (PCA), retaining the top 75 components for downstream analysis. The four samples were integrated utilizing the *harmonypy 0.0.5* python package, with convergence after two iterations. The resulting adjusted PCA matrix was used to calculate a nearest neighborhood graph with the function *sc.pp.neighbors*, with *n_neighbors* set to 15. UMAP was run using *min_dist* set to 0.05, followed by unsupervised clustering using *leiden* with *resolution* set to 1.0.

#### Cell type annotation

UniCell Deconvolve (*ucdeconvolve 0.1.2)* was utilized to perform label transfer for cell type annotations. In brief, the *allen-mouse-cortex* prebuilt reference atlas was utilized as a reference atlas to annotate cell types using the function *ucd.tl.select* with default parameters. The resulting predictions were mapped to each cell type using the function *ucd.utils.assign_top_celltypes*, with the *groupby* parameter set to a higher-resolution unsupervised leiden cluster assignment with *resolution* set to 4.0.

Following cell type assignment, top differentially expressed genes for each celltype were identified using the scanpy function *sc.tl.rank_genes_groups* with default parameters.

#### Gene set enrichment analysis

Differentially expressed genes were calculated using the scanpy function *sc.tl.rank_genes_groups*, with *groupby* set to each sample, *use_*raw set to True, and *reference* set to the control sample *VEH_NS-18-HIP*. For each non-control sample, we extracted the top 500 differentially expressed genes. Subsequently, the *gseapy 1.0.3* package was used to query *enrichr* for the *GO_Biological_Process_2021* and *GO_Molecular_Function_2021* gene sets, with *organism* set to *mus musculus.*

### Olink assay

As previously established [27], protein lysates were prepared from flash-frozen brain samples at a concentration of 1 mg/mL (*N* = 7–9 per group) using Protein Extraction Reagent (Thermo Scientific, 89900) supplemented with protease inhibitors (Thermo Scientific, 78440). Subsequently, all samples underwent analysis using the Olink^®^ Target 96 Mouse Exploratory Assay (Olink proteomics, MA, USA, 95380). Following this, the samples were sent to the Mount Sinai Human Immune Monitoring Center for assay execution and data collection. The results obtained for the analyzed protein biomarkers were provided in Normalized Protein eXpression (NPX) units.

### Statistical analysis

Statistical analysis was conducted, and all data are presented as mean ± SEM. The data were analyzed using two-way ANOVA followed by Tukey’s post hoc analysis (*p < 0.05, **p < 0.01, ***p < 0.001, ****p < 0.0001,). GraphPad Prism 8 software (GraphPad Software Inc., CA, USA) was utilized for all statistical analyses.

## Results

### Permethrin is brain-penetrant and bioavailable

**Fig. 1:**
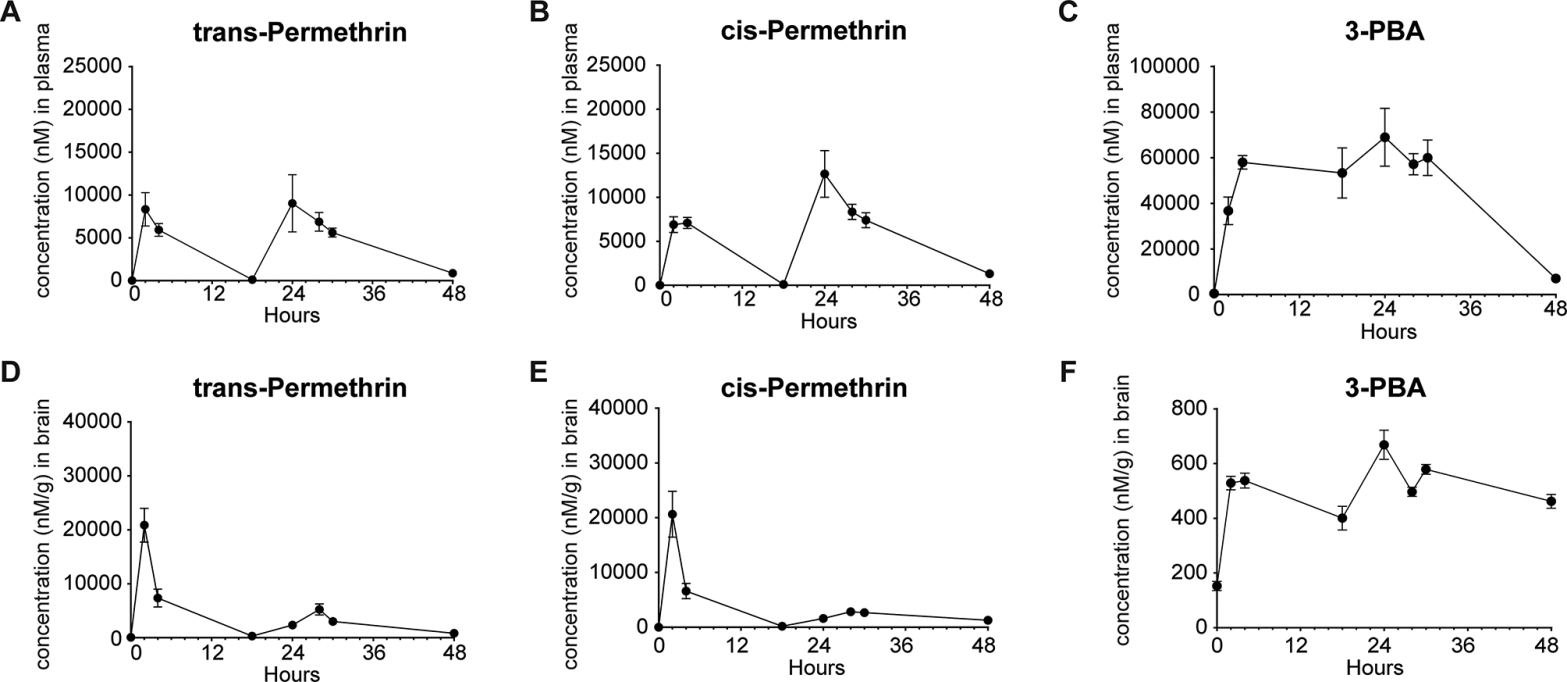
Brain and Plasma Concentrations over time. (**A**) Plasma levels of trans-Permethrin reached a maximum concentration at 24 h after acute injection (I.P.) of 200 mg/kg of permethrin. (**B**) Plasma levels of cis-Permethrin also reached a maximum concentration at 24 h. (**C**) Similarly, plasma levels of the permethrin metabolite 3-PBA reached a maximum concentration 24 h after injection. (**D**) Brain tissue levels of trans-Permethrin reached a maximum concentration at 2 h after acute injection. (**E**) Brain tissue levels of cis-Permethrin also reached a maximum concentration at 2 h after acute. (**F**) Brain tissue levels of the permethrin metabolite 3-PBA reached a maximum concentration at 24 h after acute injection. In each group, there were 5 mice, and for brain tissue, 2 replicates (separate brain hemispheres) were utilized per time point. The data are presented as means ± SEM

To confirm the permeability of permethrin through the BBB and characterize its temporal properties after exposure, we conducted pharmacokinetic analysis by evaluating parameters such as time of maximum concentration (T_max_), the maximum drug concentration (C_max_), and area under concentration-time curve (AUC). The time courses of permethrin, comprising a combination of cis- and trans-isomers, along with primary metabolite 3-phenoxybenzoic acid (3-PBA), were assessed in both plasma (Fig. 1A, B and C) and brain (Fig. 1D, E and F) over a 48-hour period following a single injection of 200 mg/kg permethrin. We first observed a “double-peak” phenomenon in both plasma and the brain at 2 h and 24 h (Fig. 1). Notably, the plasma exhibited a distinctly double-peak curve, a characteristic often associated with entero-hepatic recycling (Fig. 1A, B and C). This result indicates an increased elimination half-life, contributing to the extended action of permethrin in plasma and brain. The average plasma concentrations of cis- and trans- permethrin reached their peak at 24 h (Fig. 1A and B). Cis-permethrin displayed a C_max_ of 12655.89 ± 2646.10nM (Fig. 1B), and trans-permethrin exhibited a C_max_ of 9025.79 ± 3351.66 nM (Fig. 1A) in the plasma. In contrast, the mean brain concentrations of cis- and trans-permethrin peaked at approximately 2 h, indicating penetration of cis- and trans-permethrin into the brain occurs rapidly (Fig. 1D and E). For cis-permethrin, the mean C_max_ in the brain was 20637.85 ± 4786.71 nM (Fig. 1E), and for trans-permethrin, it was 20878.55 ± 3137.16 nM (Fig. 1D), both of which were higher than the concentrations observed in plasma. Additionally, we analyzed 3-PBA, the primary metabolite of permethrin, considering its rapid metabolism in the liver. As anticipated, a higher C_max_ (Figs. 1C and 68951.01 ± 12672.51 nM) was observed in the plasma. However, in the brain, the C_max_ of 3-PBA was found to be significantly lower than in the plasma (Figs. 1F and 668.43 ± 53.35 nM). Notably, we observed a sustained accumulation of 3-PBA in the brain parenchyma extending beyond 48 h. After reaching their peak concentrations, the mean concentration-time profiles of cis-permethrin, trans-permethrin, and 3-PBA indicated a steady decline over a period of 48 h. These findings suggest that both cis- and trans-permethrin rapidly entered the brain from periphery, achieving higher concentrations and indicating a concentrated distribution within the brain.

### Permethrin exposure primes the onset of depression-like behavior under stress through microglia activation

**Fig. 2:**
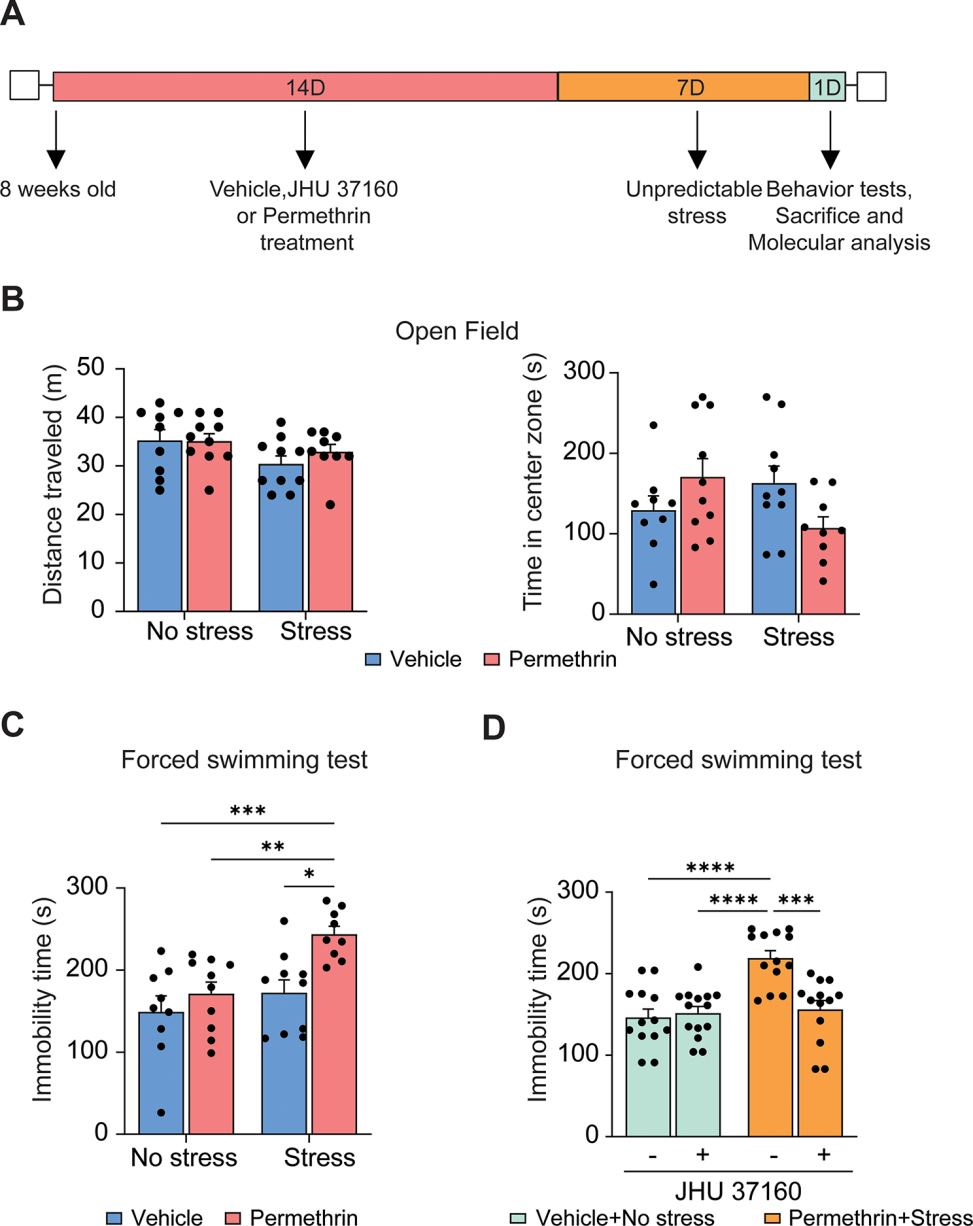
Permethrin primes stress response to induce depressive-like behavior. (**A**) Experimental scheme. (**B**-**C**) Exposure to permethrin followed by stress induced depressive (**C**) but not anxiety-like (**B**, right panel) behaviors as measured via forced swim and open field tests respectively. (**B**, left panel) Bar graphs showed locomotion activity with no alterations among all experimental groups. (**D**) Cx3Cr1^CreeEr^/hM4Di-DREADD mice expressing the Gi receptor on microglia were used to demonstrate that microglial inhibition via selective ligand JHU 37,160 is sufficient to prevent behavioral changes resulting from permethrin and stress exposure. Statistical analyses were performed using Two-Way ANOVA (*p < 0.05, **p < 0.01, ***p < 0.001, ****p < 0.001, compared to permethrin exposure followed by stress). In each group, there were13 mice and each dot represents an individual mouse. Data are expressed as the means ± SEM

To examine the effects of permethrin exposure as a priming factor, mice were exposed to permethrin (200 mg/kg) or a vehicle control for 14 days (Fig. 2A). Mice were subsequently subjected to either stress or gentle handling (no stress) for 7 days (Fig. 2A). Following our experimental protocol, anxiety-like behaviors and locomotion activity were evaluated using the OFT (Fig. 2B), while depressive-like behavior was assessed through the FST (Fig. 2C and D) to examine neuropsychological responses. There were no significant changes in the distance traveled, which is a measure of locomotion activity, or in the time spent in the center zone, which is indicative of anxiety-like behavior, across all experimental groups (Fig. 2B). In the FST, neither permethrin exposure nor stress exposure alone led to observable behavioral alterations in immobility time when compared to non-stressed mice treated with the vehicle (Fig. 2C). Interestingly, mice subjected to stress and treated with permethrin exhibited a significant increase in the time spent immobile, in comparison to all other experimental groups (Fig. 2C, *p < 0.05, **p < 0.01, ***p < 0.001). This finding suggests that exposure to permethrin acts as a priming factor in the brain, subsequently leading to depression-like behavior in mice under these specific conditions when stress is introduced.

Next, we utilized Cx3cr1^CreER/WT^: R26^LSL − hM4Di/WT^ mice to explore the impact of permethrin-induced microglia priming on the observed depression-like behavior. This mouse model is designed to selectively express Gi-inhibitory receptors (Gi-DREADDs) in microglia, controlled by the inducible Cx3cr1 promoter. Mice underwent daily treatment with 0.1 mg/kg of JHU 37160, a novel DREADD agonist known for selectively activating the Gi inhibitory signaling pathway in microglia, throughout the initial 14-day period of permethrin exposure (Fig. 2A). Following this treatment phase, the mice were then subjected to 7 days of stress, and then the FST was performed (Fig. 2A). The observed results were consistent with previous behavioral tests (Fig. 2C), indicating that mice exposed to stress and treated with permethrin displayed a notable increase in the time spent immobile (Fig. 2D, ****p < 0.0001). Notably, treatment with JHU 37160 during permethrin exposure effectively blocked depressive like-behavior in the FST (Fig. 2D, ***p < 0.01). These result indicates that microglia activation is a key mediator of the depressive-like behavior following exposure to permethrin followed by stress.

### Permethrin exposure, followed by stress, induces regional differences in the pleomorphic response of microglia cells

Pro-inflammatory microglia have been observed to undergo deramification, a process characterized by the retraction of their processes, diminished microglial complexity, and the release of inflammatory cytokines [28]. In order to further investigate the morphometric changes in microglial cells resulting from exposure to permethrin followed by stress, we conducted a detailed analysis of morphology encompassing parameters such as soma volume, branch length, the number of terminal points, and the number of intersecting segments. Additionally, we conducted a comparative analysis across the hippocampus and PFC to assess regional differences in microglial alterations.

**Fig. 3:**
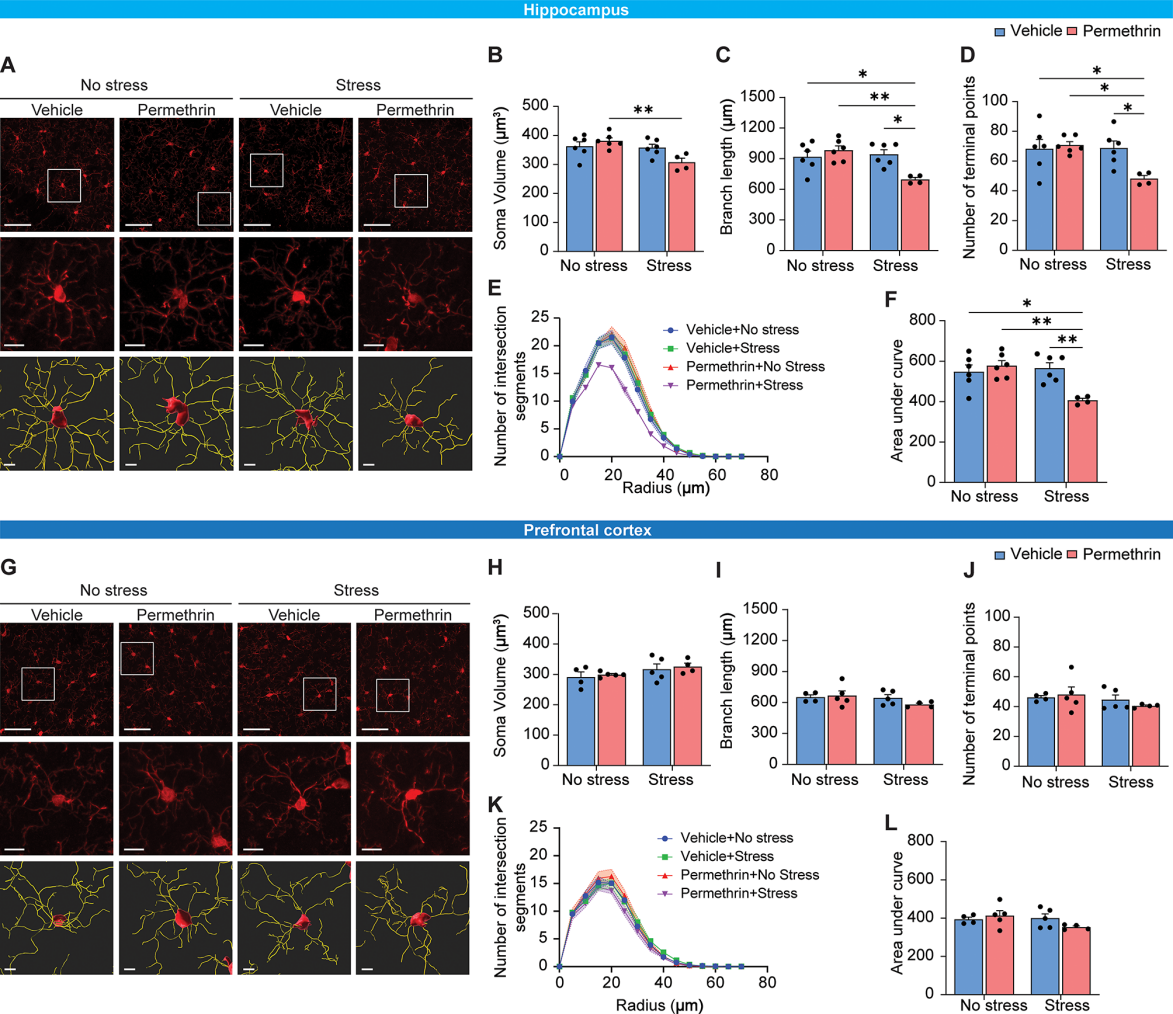
Alterations in microglial morphology induced by permethrin and stress in brain, specifically in the hippocampus prefrontal cortex. (**A**) Representative immunofluorescence images of IBA-1+ (red) cells in hippocampus. (**B**-**D**) Bar graphs represented soma volume (**B**), branch length (**C**) and number of terminal points (**D**) per microglia in hippocampus. (**E**-**F**) Sholl analysis showed that microglia in the group exposed to both permethrin and stress displayed reduced branching complexity compared to other experimental groups. (**E**) Link graph showed the quantification of the number of intersections at increasing radii, with measurements taken at 5 μm intervals. (**F**) Bar graph represented the Area Under the Curve (AUC) of the line graphs shown in (**E**). (**G**) Representative immunofluorescence images of IBA-1 + cells in prefrontal cortex. (**H**-**J**) Sholl analysis showed that microglia in the prefrontal cortex exhibited no apparent morphological changes. Bar graphs represented soma volume (**H**), branch length (**I**) and number of terminal points (**J**) per microglia in prefrontal cortex. (**K**) Link graph showed the quantification of the number of intersections in the prefrontal cortex. (**L**) Bar graph represented the AUC of the line graphs. The area outlined with a white box in the top panel is magnified in the middle panel. The bottom panel shows representative images of three-dimensional (3D) reconstructions of microglia from the middle panel Scale bars indicate 50 (top panel) and 5 (middle and bottom panel) um. Statistical analyses were performed using Two-Way ANOVA (*p < 0.05, **p < 0.01, compared to permethrin exposure followed by stress. In each group, there were 4-5mice and each dot represents an individual mouse. Data are expressed as the means ± SEM

Interestingly, the volume of microglia in hippocampus significantly decreased in mice subjected to both permethrin treatment and stress compared to mice exposed only to permethrin (Fig. 3A and B, **p < 0.01). In addition, the mice subjected to permethrin treatment and stress showed a significant decrease in the branch length of microglial processes (Fig. 3A and C, *p < 0.05, **p < 0.01), accompanied by a noticeable reduction in the number of terminal points in hippocampus (Fig. 3A and D, *p < 0.05). Sholl analysis further revealed that microglia from the group exposed to both permethrin and stress exhibited fewer and less expansive branches compared to other experimental groups (Fig. 3E). Additionally, quantification of the area under the curve further confirmed the significant decrease in microglial branching caused by permethrin and stress (Fig. 3E and F, *p < 0.05, **p < 0.01). However, no discernible morphological alterations were observed in microglia in the PFC, encompassing soma volume (Fig. 3G and H), branch length (Fig. 3G and I), terminal points (Fig. 3G and J), intersection of segments (Fig. 3G and K) and area under cover (Fig. 3G and L).

These findings underscore the importance of regional specificity in understanding neuroinflammatory responses, particularly in the hippocampus, where permethrin treatment acts as a priming factor, subsequently leading to depression-like behavior in mice under stress conditions.

### Single-cell sequencing provides profiling of distinct brain cell populations

**Fig. 4:**
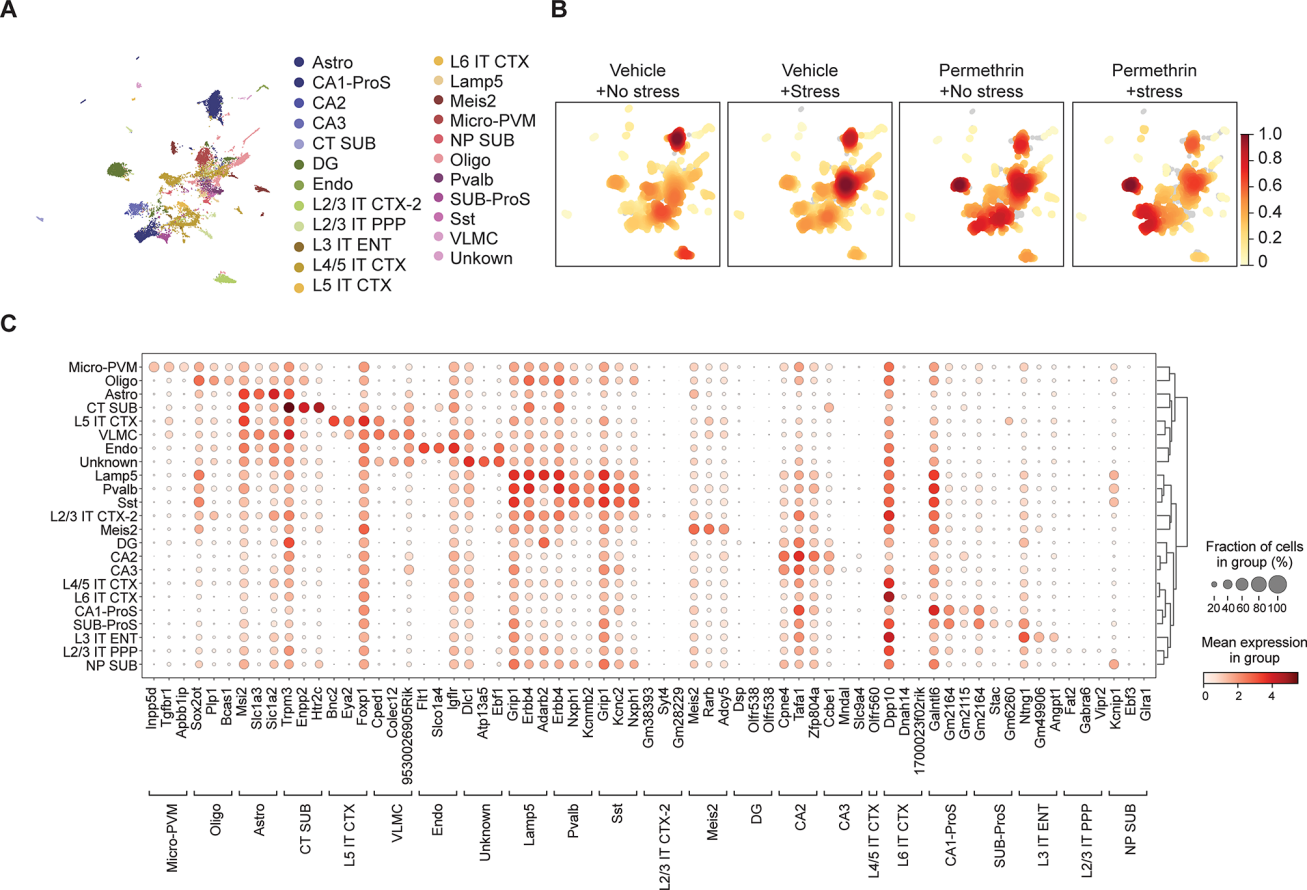
Characterization of GWI-associated brain cell population through single-cell sequencing. (**A**) UCDSelect was used to project annotations from a reference mouse cortex / hippocampus atlas onto novel dataset. Microglial cluster was confirmed by expression of canonical marker genes, including *inpp5d*,* Tgfbr1*,* Apbb1ip*. (**B**) Cell density plots for each experimental group depict a uniform distribution of cell density, with increasing intensity of red indicating specificity to the respective condition. (**C**) Differential expression analysis against predicted clusters to identify conserved markers specific to each putative cell annotation

**Fig. 5:**
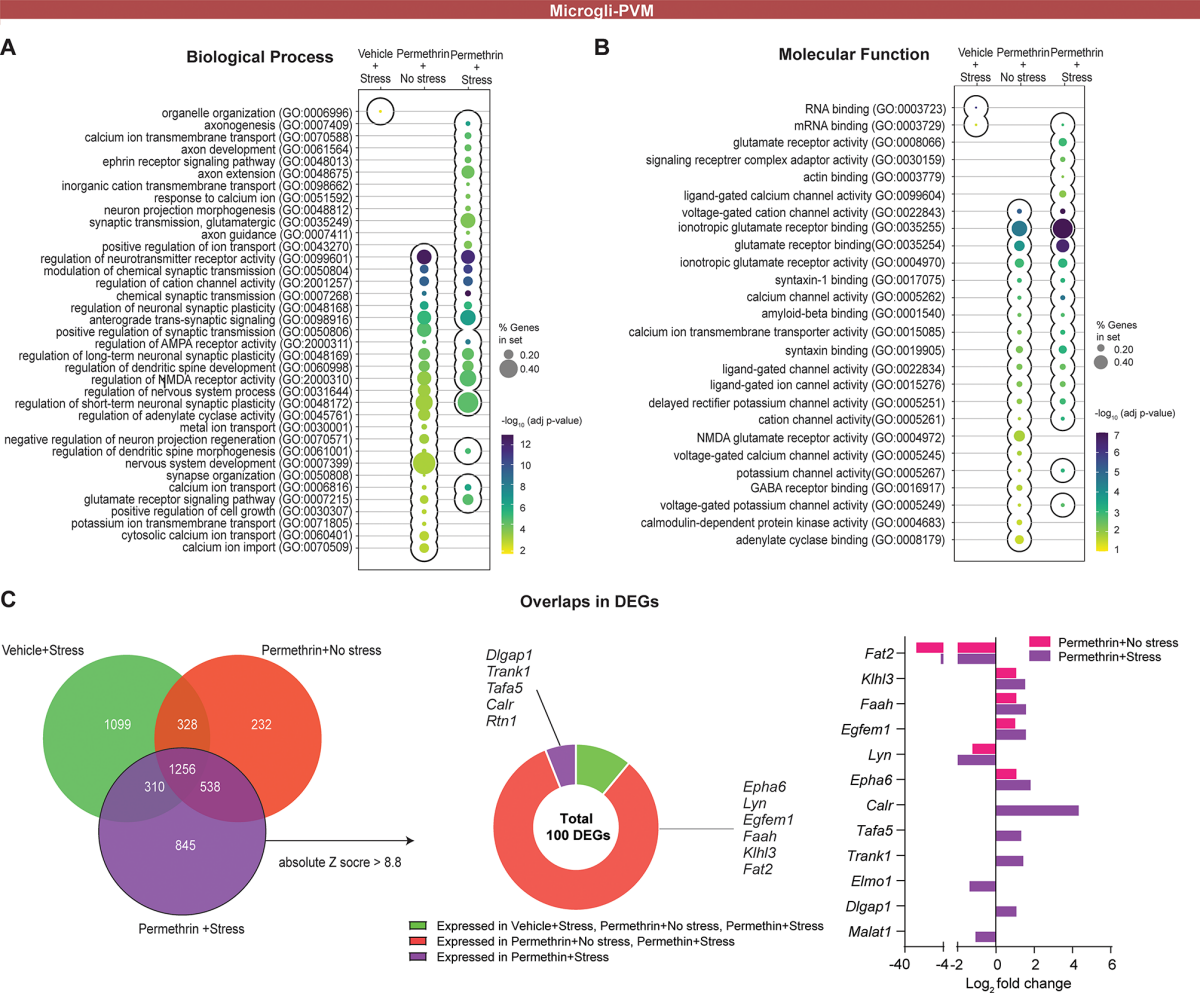
The microglia population exhibits significant enrichment in pathways linked to axon development, calcium ion transport, and neurotransmission. (**A** and **B**) Bubble plots of Gene Ontology (GO) category enrichment results in microglia cell populations for biological process (**A**) and molecular function (**B**). The color of the points reflects the − log10​ adjust p-value, with more significant p-values appearing as more intensely colored points. The size of each point corresponds to the percentage of gene sets within each GO category, with larger points indicating a higher percentage. (**C**) Venn diagram showing the number of overlapping significantly differentially expressed genes (DEGs) specifically enriched under different conditions: vehicle exposure followed by stress (green), permethrin exposure followed by no stress (red), and permethrin exposure followed by stress (pupple) (left panel). The middle panel shows the number of overlapping DEGs after sorting by an absolute z-score greater than 8.8 in the permethrin exposure followed by stress condition, and then how many genes are shared across the different experimental conditions. The right panel shows the log2 fold change values of selected DEGs expressed in the permethrin exposure followed by stress and permethrin exposure followed by no stress conditions

**Fig. 6:**
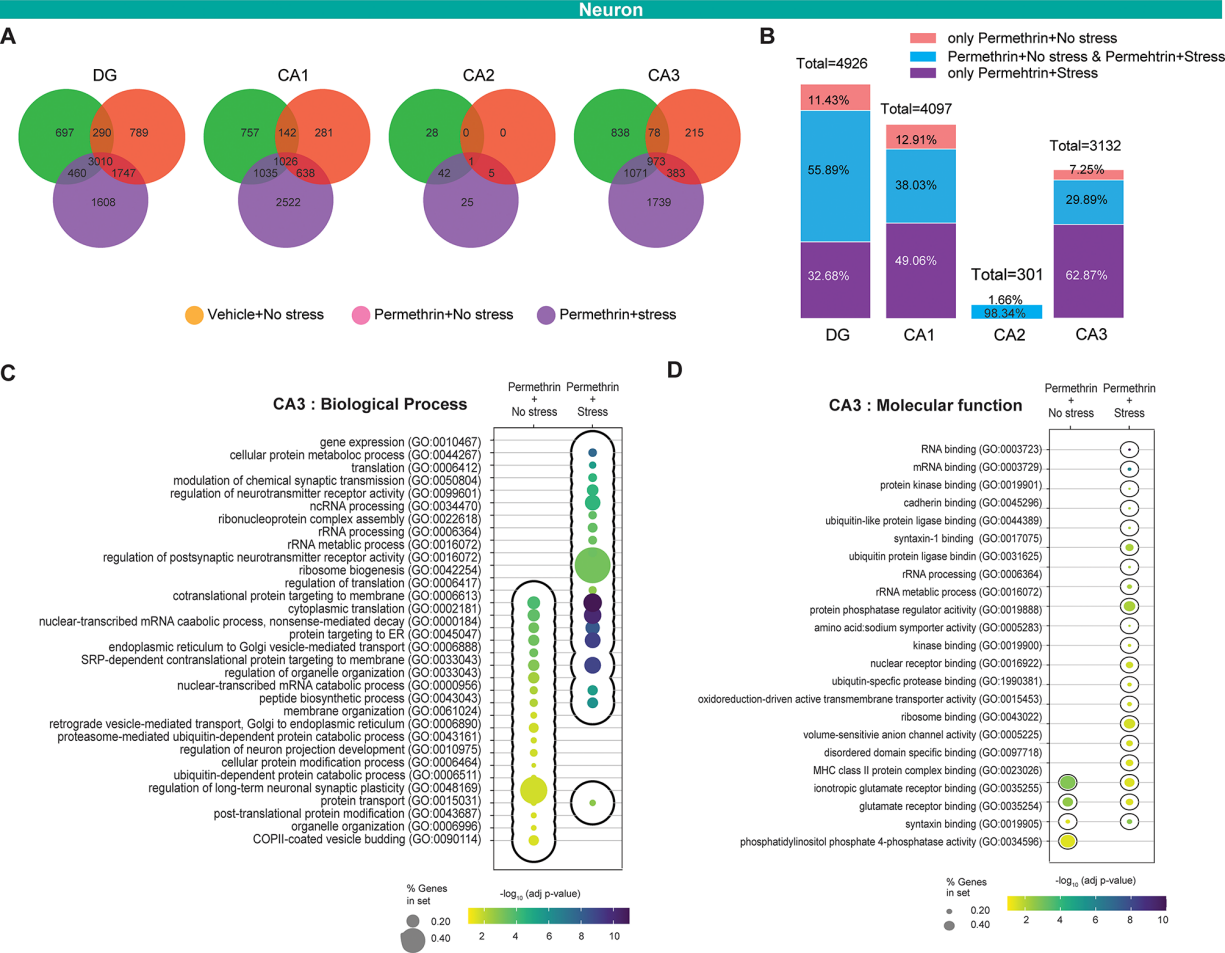
CA3 neuronal cell population exhibits significant enrichment in pathways linked to synaptic plasticity. (**A**) Venn diagram showing the number of overlapping significantly differentially expressed genes (DEGs) in DG, CA1, CA2, and CA3 regions specifically enriched under different conditions: vehicle exposure followed by stress (green), permethrin exposure followed by no stress (red), and permethrin exposure followed by stress (purple) (left panel). (**B**) Bar graphs represent the number of total genes altered and the proportion of genes represented in the Venn diagram showing the overlapping significantly DEGs within DG, CA1, CA2, and CA3. (**C** and **D**) Bubble plots of GO category enrichment results in neuronal cell populations in CA3 for biological process (**C**) and molecular function (**D**). The color of the points reflects the − log10 adjust p-value, with more significant p-values appearing as more intensely colored points. The size of each point corresponds to the percentage of gene sets within each GO category, with larger points indicating a higher percentage

To investigate whether the observed microglial morphometric changes in the hippocampus are accompanied by a genetic profile indicative of neuroinflammation, we conducted single-cell RNA sequencing analysis using 21,566 single nuclei collected from the hippocampus of mice (Figs. 4, 5 and 6).

UniCell Deconvolve Base (UCDBase) was employed to produce an initial unbiased cell type annotation, assessing cell type fractions across different conditions as previously reported [29]. Subsequently, UniCell Deconvolve Select (UCDSelect) was utilized to transfer annotations from a reference mouse cortex/hippocampus atlas onto the novel dataset (Fig. 4A) We then compared the density distribution of cell types by sample, highlighting condition-specific differences in cell type fractions post-batch correction among all experimental groups. It revealed that all experimental groups exhibited a similar density distribution, indicating a consistent profiling of distinct brain cell populations across all groups (Fig. 4B). The identity of the microglial cluster was confirmed by the expression of canonical microglia marker genes, including *inpp5d*,* Tgfbr1*,* Apbb1ip*. Additionally, the identity of neuronal cells was confirmed by the expression of canonical neuronal marker genes such as *Dsp*,* Olfr538*,* Cpne4m* for DG, *Galntl6, Gm2164, Gm2115 for CA1, Tafa1,m zfp804a *for CA2, *Cce1, Mndal, Slc9a4* for CA3 (Fig. 4C). These results confirm the identity of the identified clusters as microglia and neuronal cells, supported by the expression of canonical marker genes characteristic of each cell type using UCDBase analysis, thereby supporting the accuracy of cell type annotation.

### Transcriptome analysis reveals alterations in gene expression specific to microglia following exposure to permethrin, followed by stress

To elucidate the transcriptional networks impacted within microglia populations associated with depression-like behavior in mice exposed to permethrin and/or stress, we conducted a functional enrichment analysis, encompassing biological process (Fig. 5A) and molecular function (Fig. 5B). In Fig. 5A, we compared the biological processes selected based on adjusted p-values of < 0.05. While minimal changes were evident in the stress group, both permethrin exposure alone and in combination with stress showed similar alterations in biological processes. Notably, the permethrin with stress condition exhibited heightened significance and involvement of a greater number of gene sets in the same biological processes compared to permethrin-only group. Specifically, microglia cell population in permethrin with stress group displayed enriched biological processes associated with neuronal development (axon development, axon guidance, and the ephrin receptor signaling pathway), neuronal communication (calcium signaling, synaptic transmission, and glutamate receptor activity), as well as neuronal morphogenesis. In the assessment of molecular functions (Fig. 5B), a consistent trend emerged where minimal alterations were observed in the stress group, whereas both the permethrin-only and permethrin combined with stress groups exhibited similar patterns. Notably, within the permethrin with stress group, distinct molecular activities were noted, including ionotropic glutamate receptor activity and ligand- or voltage-gated calcium channel activity. These findings underscore parallels in biological processes concerning neuronal signaling, ion channel modulation, and synaptic transmission.

Subsequently, we investigated differentially expressed genes (DEGs) to discern variations between permethrin treatment alone and permethrin treatment with stress. In Fig. 5C, notable modifications in gene expression were observed, with 2,949 genes in the permethrin and stress group and 2,354 genes in the permethrin-only group exhibiting significant alterations compared to the vehicle group without stress (adjusted p-value < 0.05, Fig. 5C, left). Moreover, 232 genes were exclusively altered in the permethrin-only group, while 845 genes uniquely changed in the permethrin and stress group, suggesting that the permethrin with stress group exhibited more significant gene alterations. Following, utilizing the absolute Z score, we identified the top 100 genes in the permethrin with stress group (Fig. 5C, middle). It was observed that only 5 genes such as *Dlgap1*,* Trank1*,* Tafa5*,* Calr*,* Rtn1* were exclusively expressed in the permethrin with stress group (Fig. 5C, right). Additionally, 87 genes showed differential expression in both groups, with the permethrin with stress group exhibiting a significantly more pronounced increase or decrease in expression compared to the permethrin-only group. Notably, 6 genes such as *Epha6*,* Lyn*,* Egfem1*,* Faah*,* Klhl3 and Fat2* displayed the most substantial changes, with their expression levels in the permethrin with stress group being 1.4-fold higher or lower than those observed in the permethrin-only group (Fig. 5C, right). Overall, the results highlight the potential synergistic impact of permethrin and stress on microglia gene expression, which could contribute to the underlying mechanisms involved in depression-like behavior observed in the animal model (Fig. 2).

### Permethrin exposure followed by stress induces a significant alteration in gene expression patterns across regionally distinct neuronal populations

To investigate how permethrin exposure followed by stress impacts transcriptional networks in neuronal cells, which functions are potentially mediated by microglia, in hippocampal regions including CA1, CA2, CA3, and DG, we first compared the number of DEGs across groups: permethrin exposure followed by no stress, permethrin exposure followed by stress, and vehicle exposure followed by stress (Fig. 6A and B). Adjust p-value threshold of 0.05 was used to identify DEGs in CA1, CA2, CA3, and DG of the hippocampus in mice from each group. Interestingly, permethrin exposure followed by stress displayed a significantly higher number of DEGs compared to the other groups in all hippocampal regions (Fig. 6A). Analysis of hippocampal regions CA1, CA2, CA3, and DG following permethrin exposure and subsequent stress revealed a prominent increase of DEGs, with CA1 exhibiting the most notable rise compared to other regions, followed by CA3. (Fig. 6A). Given the observed differences in microglial alterations between the permethrin-exposed groups with and without stress (Fig. 5), we specifically compared gene expression in these groups. Among hippocampal regions, only CA3 region exhibited a high percentage of unique expression in DEGs in the permethrin exposure followed by stress group. 62.87% were unique to the permethrin with stress group, while 29.89% were common to both permethrin exposed groups (with or without stress), and only 7% were specific to the permethrin without stress group (Fig. 6B).

To further explore the functional implications of the observed gene expression alterations in CA3 region, we conducted Gene Ontology (GO) enrichment analysis, specifically comparing the permethrin exposure followed by no stress and permethrin exposure followed by stress groups (*p* < 0.05, Fig. 6C and D). Compared to the permethrin exposure followed by no stress group, the permethrin exposure followed by stress group in CA3 region showed significantly enriched GO terms (Fig. 6C) related to signal transduction, including modulation of chemical synaptic transmission, regulation of neurotransmitter receptors, and regulation of postsynaptic neurotransmitter receptor activity. Notably, even for shared biological processes, permethrin with stress group displayed a greater number of genes and a more significant level of enrichment. We then focused on the molecular function of DEGs using GO enrichment analysis (*p* < 0.05, Fig. 6D). GO enrichment analysis of DEGs revealed a significant enrichment for molecular function terms (*p* < 0.05, Fig. 6D). The permethrin exposure followed by stress group exhibited a notably greater abundance of enriched terms compared to the permethrin exposure followed by no stress group, including synaptotagmin-1 binding, amino acid sodium symporter activity, and volume-sensitive anion channel activity. (Fig. 6D). Overall, this results indicate that permethrin exposure followed by stress triggered a prominent rise in differentially expressed genes, particularly in CA1 and CA3, with CA3 region exhibiting distinct patterns suggestive of disrupted neuronal communication.

### Exposure to permethrin followed by stress altered protein expression associated with neuroinflammation and synaptic plasticity

**Fig. 7:**
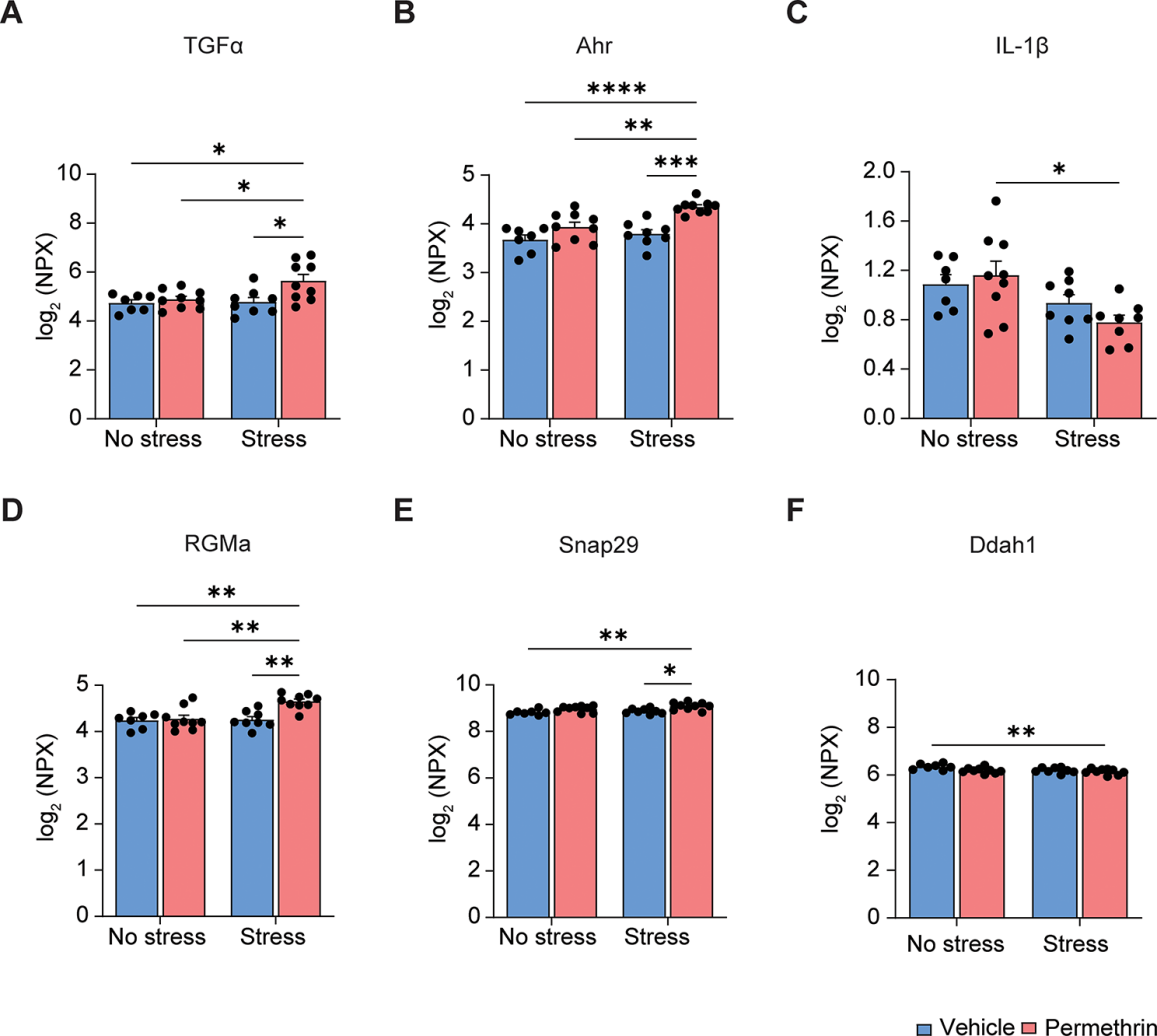
Olink proteomic analysis in the GWI mouse model. (**A**-**F**) Box graphs represented significant changes in protein expression levels of TGFα (**A**), Ahr (**B**), IL-1β (**C**), RGMa (**D**), Snap29 (**E**), and Ddah1 (**F**) in the hippocampus across all experimental groups. Statistical analyses were performed using Two-Way ANOVA (*p < 0.05; **p < 0.01, ***p < 0.001; ****p < 0.0001; compared to the permethrin followed by stress group, *n* = 7–9 mice for each group). Data are expressed as the means ± SEM

To investigate the proteomic alterations in mice subjected to permethrin exposure followed by stress, we examined the abundance of a specific set of 92 proteins associated with essential biological functions using proximity extension assay-based Olink technology. We identified significant alterations in the levels of six proteins specifically within the hippocampi of mice exposed to permethrin followed by stress (Fig. 7).

Specifically, there was a notable increase in Transforming growth factor alpha (TGF-α) in mice exposed to permethrin followed by stress compared to other experimental groups (*p < 0.05, Fig. 7A). Additionally, Aryl hydrocarbon receptor (Ahr), known for its involvement in regulating microglial activation and neuroinflammation, showed elevated levels in the same mice (****p < 0.0001, ***p < 0.001, **p < 0.01, Fig. 7B). Concurrently, there was a decrease in the expression of Interleukin-1beta (IL-1β) (*p < 0.05, Fig. 7C).

Repulsive guidance molecule A (RGMa), renowned for its capacity to inhibit neurite growth and newborn neuron survival in the adult dentate gyrus, demonstrated notable upregulation in the permethrin followed by stress group compared to the other experimental groups (**p < 0.01, Fig. 7D). Additionally, Synaptosomal-associated protein 29 (Snap-29), responsible for impeding SNARE complex disassembly and thereby diminishing synaptic transmission, exhibited heightened expression in the permethrin followed by stress group relative to both the no stress and stress alone groups (**p < 0.01, *p < 0.05, Fig. 7E). Conversely, N(G), N(G)-dimethylarginine dimethylaminohydrolase 1(Ddah1), a hydrolase responsible for regulating genes associated with the synthesis and transportation of acetylcholine, exhibited significant decrease in expression in the permethrin followed by stress group (**p < 0.01, Fig. 7F). These findings highlight the multifaceted effects of permethrin exposure followed by stress on both inflammatory responses and synaptic plasticity within the hippocampi of mice, underscoring the complex interplay between microglia activation and neuronal function.

## Discussion

GWI is a complex multi-symptom disorder affecting approximately one third of veterans who served in the first GW [30, 31]. These soldiers were exposed to various pesticides, including permethrin, DEET, and chlorpyrifos, as well as chemical weapons, smoke from oil well fires, and fine particulate matter from sandstorms [32]. Upon returning from combat, affected veterans experienced a range of symptoms such as fatigue, anxiety, gastrointestinal distress, and cognitive and psychiatric disturbances [4]. High levels of stress reported by many veterans during their service suggest that physical and psychological stress may have synergized with toxin exposure [3]. Currently, there is no approved therapeutic for GWI, with most treatments focusing on symptom management [33]. Although the exact cause of GWI has not been identified, permethrin exposure is suspected to play a role [34]. Understanding the mechanistic factors involved and developing appropriate animal models is crucial for advancing care for this underserved population. Thus, the mouse model used in our current studies to examine interactions between environmental toxin exposure and mild stress could serve as a valuable model for studying GWI. This model may help elucidate the underlying mechanisms and contribute to the development of targeted treatments for veterans suffering from GWI.

The exposome, encompassing the cumulative impact of stressors and environmental exposures experienced throughout an individual’s life, is an emerging area of research with profound implications for neuropsychological health [35]. In particular, interactions between various low-level or mild exposures represent a potential hazard which may go un-noticed due to the seemingly innocuous effects of individual low-level exposures [36]. In the present study, we investigated the effects of low-level permethrin exposure at levels below the threshold required for acute intoxication for their potential synergistic interactions with 7 days of mild stress exposure. While chronic stress over a 30 day period is associated with psychiatric and changes in mice, 1 week of stress has not been shown to have neuroinflammatory or behavioral effects [23]. We revealed a significant increase in depression-like behavior in mice exposed to both permethrin and stress compared to those exposed to either factor alone or controls (Fig. 2). This suggests that permethrin exposure acts as a priming factor, sensitizing the brain to subsequent stressors and exacerbating depressive symptoms. Exposure to permethrin triggers a rapid influx of sodium ions into microglia cells, leading to increased intracellular sodium accumulation; prolonged exposure for 24 h activates microglia and results in neuroinflammation by releasing pro-inflammatory cytokines [13]. In the exploration of microglial functions within the CNS, chemogenetic G protein-coupled receptors (GPCRs), including Gi-signaling (e.g., hM4Di) and Gq-signaling (e.g., hM3Dq) DREADDs, have previously been utilized as sophisticated tools for understanding microglial processes [37]. Importantly, our chemogenetic modulation experiments via microglia expressing DREADD receptors highlight the pivotal role of microglial activation in mediating this depressive-like behavior, as blockade of microglial activation effectively mitigated the observed behavioral changes (Fig. 2). Thus, the ability of permethrin to stimulate neuroinflammation through microglia underscores its potential as a priming factor in enhancing susceptibility to depression.

Consistent with previous research [34], our results demonstrate that permethrin, including both cis- and trans- isomers, is capable of crossing the blood-brain barrier, but reaches higher concentrations in the plasma (Fig. 1). This finding highlights the potential neurotoxicity of permethrin, given its ability to directly interact with CNS components. Exposure to permethrin initiates a rapid and continuous influx of sodium into microglial cells through voltage-gated sodium channels (VGSC) [13]. Consequently, there is an aberrant accumulation of intracellular sodium, leading to the activation of microglia and the subsequent upregulation of pro-inflammatory cytokine such as tumor necrosis factor alpha (TNFα [13]. This pro-inflammatory response is characterized by deramification, a phenomenon defined by the retraction of microglial processes [38]. To elucidate these morphometric changes, a detailed analysis encompassing soma volume, branch length, terminal points, and intersecting segments in microglia was conducted (Fig. 3). Hippocampus and PFC engage in a bidirectional interaction that influences cognitive functions and the processing of emotional information [39]. Functional imaging studies in depression patients have identified significant abnormalities in the structure, activation, and functional connectivity of the hippocampal–PFC circuit [40]. Specifically, the hippocampus and PFC demonstrate significant changes in microglial morphology in response to various stressors [34, 36, 37]. We revealed significant reductions in microglial volume, branch length, and number of terminal points in the hippocampus of mice exposed to permethrin, followed by stress. We also further confirmed a decrease in microglial branching complexity in the hippocampus following combined exposure through sholl analysis. In contrast, no significant morphological alterations were observed in microglia within the PFC across all parameters analyzed. The observed deramification and reduced complexity of microglia in the hippocampus suggest a potential mechanism underlying the development of depression-like behavior following exposure to permethrin and stress.

We investigated the morphology of microglia in the hippocampus and PFC based on the crucial role of these distinct regions in regulating mood and emotional behavior [39, 40]. We observed significant alterations in hippocampal microglial morphology, which may contribute to the development of depressive-like behavior after exposure to permethrin followed by stress. Our study investigated the relationship between microglial changes in the hippocampus and depression-like behavior in order to develop valuable insights into the mechanisms underlying the link between neuroinflammation and mood disorders within the context of GWI. Specifically, our findings showed that morphological alterations are accompanied by transcriptional changes indicative of neuroinflammation, as revealed by single cell RNA sequencing analysis (Figs. 4, 5 and 6). A recent study indicates that UCDBase was established to leverage the entirety of publicly available scRNA-Seq data to construct a unified training corpus for deep learning [29]. In addition, UCDSelect complements UCDBase by enabling context-specific deconvolution through transfer learning of UCDBase features, incorporating user-defined cell signatures for tailored analyses [29]. Our analysis validated the identified clusters as microglia and neuronal cells through the cell type-specific marker gene (Fig. 4). Functional enrichment analysis indicates a synergistic impact of permethrin and stress on microglia gene expression, with the stress-only group showing minimal changes, while the permethrin-only group exhibits a pattern similar to the permethrin-with-stress group, which displays more pronounced alterations (Fig. 5). Specifically, we found that among the top 100 genes, only five, such as *Dlgap1*,* Trank1*,* Tafa5*,* Calr*, and *Rtn1*, were exclusively altered in the permethrin exposure followed by stress group. *Dlgap1*, *Trank1*, and *Tafa5* are established risk genes for neuropsychiatric diseases, with *Dlgap1* specifically related to major depressive disorder, *Trank1* associated with bipolar disorder, and *Tafa5* linked to depressive-like behaviors [41–43]. Calr is released by microglia following exposure to lipopolysaccharide [44]. This extracellular calreticulin was observed to induce chemoattraction and activation of microglia, leading to the release of pro-inflammatory cytokines TNF-α, IL-6, and IL-1β, as well as the chemokine (C-C motif) ligand 2 [44]. In addition, Calr serves as an “eat-me” signal, promoting the engulfment of cells by phagocytes through the interaction with the low-density lipoprotein receptor-related protein 1 [45]. These findings suggest that these five genes may play a role in modulating microglial activity and contributing to the inflammatory response in the brain, potentially leading to depression-like behavior associated with permethrin exposure as a priming factor. Future research in depth is needed to elucidate the roles of these genes in microglial modulation and neuroinflammation, as well as their contribution to depression-like behavior associated with permethrin exposure.

In this study, we identified significant changes induced within the microglial cell population in response to exposure to permethrin followed by stress. We also identified changes in the expression of genes genes associated with neuronal development, neuronal communication, and neuronal morphogenesis (Fig. 5) via functional analysis. Microglia exert a critical role in maintaining synaptic integrity throughout development and adulthood via dynamic sculpting of the synapse [46]. Aberrant microglial function in the mature brain has been implicated in pathological synaptic loss and dysfunction associated with neuroinflammation, and depression [47, 48]. For instance, in depressed patients, the reduction in synapse number and function is linked to microglial engulfment of synapses [49]. Depressive-like behaviors observed in several chronic stress models have been linked to the specific removal of postsynaptic dendritic spines in targeted branches, along with atrophy of neurons [50]. To investigate how permethrin exposure followed by stress affects transcriptional networks in different types of neuronal cells, we focused on the hippocampal subregions (CA1, CA2, CA3, and DG). Our analysis revealed a significant increase in DEGs in neurons within these regions after permethrin exposure and subsequent stress. Notably, CA1 showed the most significant increase in DEGs, followed by CA3 (Fig. 6). The microglia density in CA3 region of the mouse hippocampus is higher than in both CA1 region and DG [51, 52]. CA1 region has a higher microglia density compared to DG [52]. It has been proposed that this variation in microglial density could influence site-specific vulnerability in the hippocampus and that the uneven distribution of microglia may play a role in regulating hippocampal neuronal activity. These findings suggest that the high density of microglia in CA3 region might be a key factor in the significant alterations in neuronal DEGs observed there following permethrin and stress exposure. Further research is needed to specifically examine how microglia-neuron interactions in the CA3 region are affected by permethrin exposure followed by stress, and to elucidate the mechanisms driving these changes.

Consistent with these findings, our proteomic analysis uncovers alterations in key proteins involved in neuroinflammation and synaptic plasticity within the hippocampi of mice exposed to permethrin followed by stress (Fig. 7). Notably, we found the upregulation of pro-inflammatory cytokines such as TGFα and Ahr, underscoring the dysregulated inflammatory response elicited by permethrin exposure and stress (Fig. 7). The upregulation of TGF-α has been implicated in the activation of microglia, which are key players in neuroinflammatory processes and may contribute to neuronal damage by activating microglia [53]. AhR is a critical mediator for peripheral immune functions involving the differentiation of dendritic cells and the tissue-specific proinflammatory gene expression [54, 55]. In the brain, AhR is ubiquitously expressed in areas including the cerebral cortex, hippocampus, and cerebellum [56], and is related to its environmental ligands-associated sensorimotor and cognitive abnormalities based on its aggravation of oxidative stress or excitotoxicity in neurons [57]. AhR can also directly activate cytokine transcription and AhR increases NOX activity by increasing the expression of p47phox to elicit oxidative stress, which contributes to cytokine transcription such as TNF- α [58]. Additionally, we found that the expression of synaptic plasticity protein molecules (RGMa, SNAP-29, and Ddah1) in response to permethrin followed by stress (Fig. 7). SNAP-29 serves as a negative modulator by slowing the disassembly of the SNARE complex and consequently diminishing synaptic transmission in cultured neurons. This effect is associated with the attenuation of both the recycling process of the SNARE-based fusion machinery and the turnover of synaptic vesicles [59]. RGMa has been identified to hinder synapse formation by disrupting the expression of presynaptic protein synapsin-1 and postsynaptic protein PSD-95 in cortical neurons. [60]. Recent study suggests that RGMa regulates neuronal branching through the RhoA pathway, thereby impacting synaptic plasticity. [61]. RGMa can also inhibit axon growth in CNS through its potential suppression of axon growth by inducing the expression of Rho-associated coiled-coil protein kinase in neurons. [62]. Additionally, research indicates that mice subjected to an Mg-restricted diet display depression-like behavior and diminished expression of DDAH1, the enzyme responsible for breaking down N, N dimethyl-L-arginine, a significant competitive inhibitor of nitric oxide synthase [63].

In conclusion, our study demonstrates that permethrin exposure followed by stress induces depression-like behaviors in mice through microglial activation and upregulation of pro-inflammatory cytokines, particularly in the hippocampus. Additional alterations in gene expression, observed through sc sequencing, suggest potential disruptions in neuronal communication mediated by microglial activation. To gain a more thorough understanding of how microglial activation affects specific neuronal communication pathways, further in-depth studies are needed to validate and explore the key molecules or mechanisms identified in this research. Our findings highlight the complexity of GWI pathology, revealing how exposure to chemical toxins and psychological stress during the GW may have produced a pronounced neuroinflammatory response and co-inciding psychiatric symptoms. This complex interaction underscores the need to consider both chemical exposures and psychological stress in understanding the full scope of GWI, which may inform more targeted interventions and treatments for affected individuals.

## Electronic supplementary material

Below is the link to the electronic supplementary material.

Supplementary Material 1: Supplementary Fig. 1. Quality Control Metrics for snRNA-Seq Samples. (A-C) Violin plots with overlayed scattered strip plots of pre-filtered single-nuclei grouped by individual sample (x-axis), with respect to the percent of total counts per cell originating from either mitochondrial (A), hemoglobin (B), or ribosomal (C) genes (y-axis). (D) Scatterplot colored by individual sample showing individual single-nuclei arranged by total number of gene counts (x-axis) with respect to the total number of unique genes identified with at least one count per nucleus (y-axis). (E) Violin plots with overlayed scattered strip plots of pre-filtered single-nuclei grouped by individual sample (x-axis), with respect to the log-transformed total number of genes counts per nucleus (y-axis). (F) Violin plots with overlayed scattered strip plots of pre-filtered single-nuclei grouped by individual sample (x-axis), with respect to the log-transformed total number of unique genes identified with at least one count per nucleus (y-axis)

## Data Availability

No datasets were generated or analysed during the current study.
