# Supplementary figures and images for: Permethrin exposure primes neuroinflammatory stress response to drive depression-like behavior through microglial activation in a mouse model of Gulf War Illness
Source: J Neuroinflammation. 2024 Sep 13;21:222. doi: 10.1186/s12974-024-03215-3 (PMC11396632; doi:10.1186/s12974-024-03215-3)

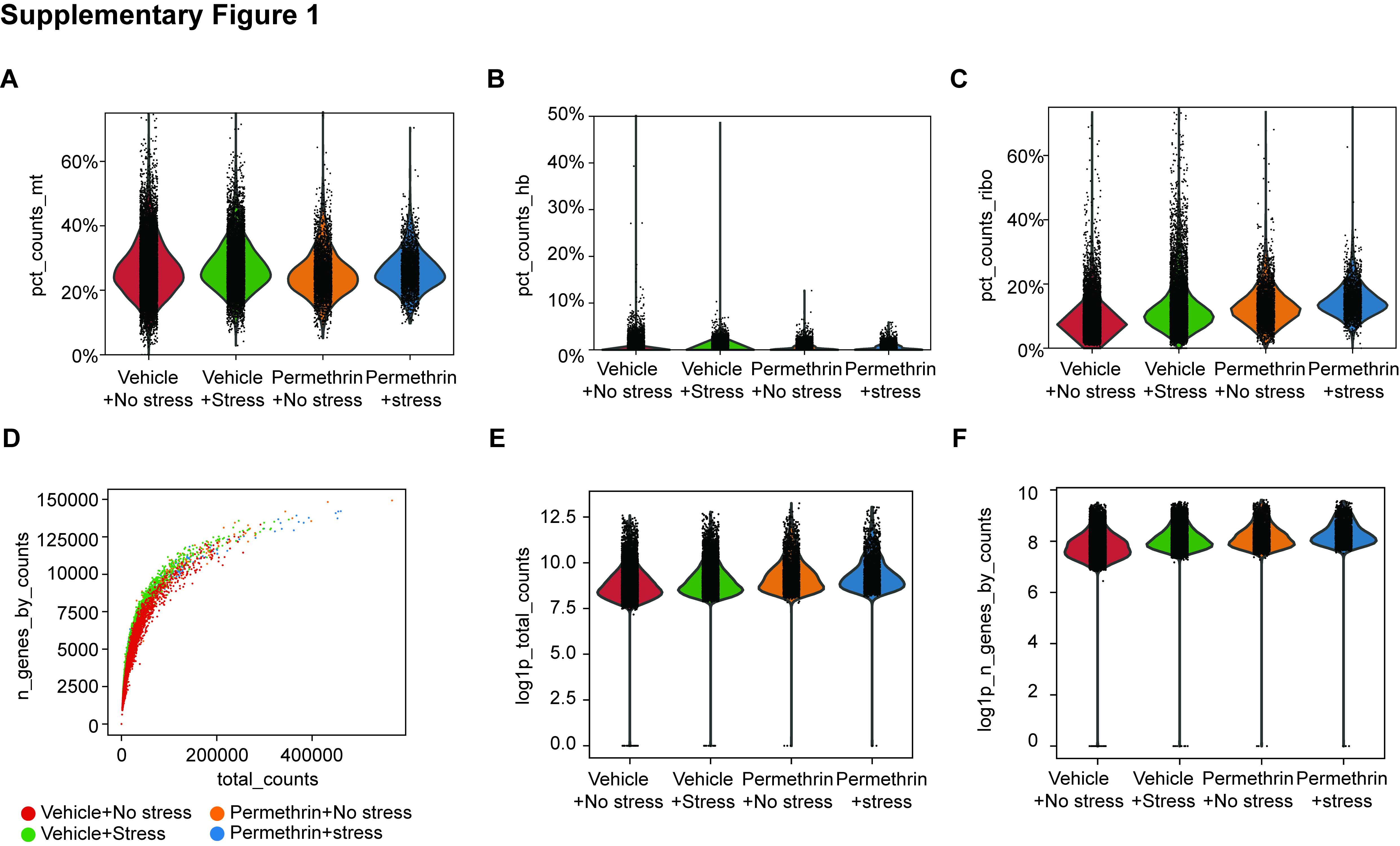

Supplement: Supplementary file 1 — Supplementary Material 1: Supplementary Fig. 1. Quality Control Metrics for snRNA-Seq Samples. (A-C) Violin plots with overlayed scattered strip plots of pre-filtered single-nuclei grouped by individual sample (x-axis), with respect to the percent of total counts per cell originating from either mitochondrial (A), hemoglobin (B), or ribosomal (C) genes (y-axis). (D) Scatterplot colored by individual sample showing individual single-nuclei arranged by total number of gene counts (x-axis) with respect to the total number of unique genes identified with at least one count per nucleus (y-axis). (E) Violin plots with overlayed scattered strip plots of pre-filtered single-nuclei grouped by individual sample (x-axis), with respect to the log-transformed total number of genes counts per nucleus (y-axis). (F) Violin plots with overlayed scattered strip plots of pre-filtered single-nuclei grouped by individual sample (x-axis), with respect to the log-transformed total number of unique genes identified with at least one count per nucleus (y-axis) [file 12974_2024_3215_MOESM1_ESM.tif]
